# Implementation of a Virtual Reality-Based Program for Fall Risk Reduction in Older Adults in Primary Health Care

**DOI:** 10.3390/ijerph23040504

**Published:** 2026-04-15

**Authors:** Sebastián Burgos-Carrasco, Yislem Barrientos-Cabrera, Valentina Rivera-Mora, Laura Martínez-González, Bryan Arpe-Hernández, Consuelo Cruz-Riveros, Diego Fernández-Cárdenas, Iván Yañez-Cifuentes, Roberto López-Andaur

**Affiliations:** 1 Académico Escuela de Terapia Ocupacional, Facultad de Salud y Ciencias Sociales, Universidad de Las Américas, Concepción 4030000, Chile; 2 Académica Escuela de Fonoaudiología, Facultad de Salud y Ciencias Sociales, Universidad de Las Américas, Concepción 4030000, Chile; ybarrientos@udla.cl; 3 Escuela de Enfermería, Facultad de Salud, Universidad Santo Tomás, Concepción 4030000, Chile; vmora15@santotomas.cl; 4 Académica Escuela de Terapia Ocupacional, Facultad de Salud, Universidad Santo Tomás, Concepción 4030000, Chile; lmartinez10@santotomas.cl; 5 Académico Carrera Terapia Ocupacional, Facultad de Medicina y Ciencias de la Salud, Universidad Central de Chile, Santiago 8320000, Chile; bryan.arpe@ucentral.cl; 6 Académica Escuela de Enfermería, Facultad de Salud y Ciencias Sociales, Universidad de Las Américas, Concepcion 4030000, Chile; ccruz@udla.cl; 7 School of Occupational Therapy, Health Sciences Faculty, Catholic University of Maule, Talca 3530000, Chile; dfernandezc@ucm.cl; 8 Escuela de Terapia Ocupacional, Facultad de Ciencias de la Salud, Universidad Autónoma de Chile, Santiago 7500912, Chile; ivan.yanez@uautonoma.cl; 9 Exercise and Rehabilitation Sciences Institute, School of Occupational Therapy, Faculty of Rehabilitation Sciences, Universidad Andres Bello, Santiago de Chile 4030000, Chile; roberto.lopez@unab.cl

**Keywords:** virtual reality, immersive virtual reality, exergames, program exercises, older person, risk of falls, frailty, program therapeutic

## Abstract

**Highlights:**

**Public health relevance—How does this work relate to a public health issue?**

- Population aging in Latin America and the Caribbean is associated with an increased prevalence of frailty, functional decline, and fall risk, representing a major public health concern due to its impact on morbidity, mortality, dependency, and healthcare utilization.
- Falls among older adults are one of the leading causes of disability, hospitalization, and loss of autonomy, generating a substantial burden on healthcare systems, particularly at the primary care level.

**Public health significance—Why is this work of significance to public health?**

- This study proposes an innovative intervention based on immersive virtual reality for dual-task training, simultaneously addressing motor and cognitive components that are critical for fall prevention and the maintenance of functional capacity in older adults.
- The implementation of the protocol within a community-based primary care setting allows for the evaluation of its feasibility under real-world conditions, contributing to the generation of applicable and scalable evidence for public health systems.

**Public health implications—What are the key implications or messages for practitioners, policy makers and/or researchers in public health?**

- For healthcare professionals and policy makers, this study provides evidence on the potential integration of accessible technologies, such as virtual reality, into fall prevention programs and strategies aimed at promoting autonomy in older adults, supporting more engaging and personalized interventions.
- For public health research, the findings may inform future randomized controlled trials and the development of cost-effective intervention models, while addressing critical factors such as the digital divide, adherence, and implementation in community-based settings.

**Abstract:**

Aging is a progressive and heterogeneous biological process influenced by multiple factors that may compromise physical and cognitive capacities and increase the risk of frailty, functional decline, and falls in older adults. Falls represent a major public health concern due to their impact on independence and long-term care demand. Immersive virtual reality (IVR) delivered through active video games (exergames) has emerged as a preventive strategy that integrates sensory, motor, and cognitive stimulation within controlled and engaging environments, particularly where traditional programs face challenges related to adherence and individual adaptation. This study aims to determine the feasibility and implementation of an IVR-based program for falls prevention in older adults at risk of frailty in primary health care (PHC). A quasi-experimental pre–post design will be conducted with an intervention group (IVR/exergames) and a conventional control group, including a total sample of 40 participants (20 per group). The protocol comprises three phases: baseline assessment and IVR familiarization; a 12-week intervention delivered twice weekly; and post-intervention assessment. The primary outcome will be fall risk assessed using the Timed Up and Go (TUG) test. Secondary outcomes include physical performance (Short Physical Performance Battery, SPPB, and handgrip dynamometry) and psychological aspects related to falls (Falls Efficacy Scale International, FES-I, and Activities-specific Balance Confidence Scale, ABC). Feasibility indicators will include recruitment, adherence, retention, and cybersickness. A reduction in TUG time is expected, providing preliminary evidence on the feasibility of integrating IVR-based programs for falls prevention within PHC systems.

## 1. Introduction

According to the World Health Organization (WHO), aging is a progressive biological process associated with the accumulation of molecular and cellular damage across the life course, leading to a gradual decline in physical and cognitive capacities [1,2]. This process does not manifest uniformly across individuals, as aging trajectories and their progression are strongly influenced by the interaction of risk and protective factors accumulated over time [3]. Globally, it is projected that by 2030, one in six people will be aged 60 years or older, reflecting a profound demographic shift with significant implications for health and social protection systems [4]. In Latin America and the Caribbean, this demographic transition presents specific characteristics. According to United Nations estimates, by 2022, the region was experiencing a more accelerated population aging process compared with other world regions [5]. In this context, countries such as Uruguay and Chile are expected to be among the most aged in South America by 2030, posing substantial public health challenges in terms of functionality and quality of life among older adults [6]. In Chile, this transition is occurring at a particularly rapid pace, with 14% of the population aged 65 years or older and a life expectancy exceeding 81 years [7,8].

The increase in the average age of the population has been associated with a higher prevalence of chronic diseases and geriatric syndromes, particularly within a context characterized by high levels of sedentary behavior. This situation contributes to the development of conditions such as frailty, sarcopenia, and balance disorders, which represent key determinants of functional decline and loss of autonomy in older adults [9,10]. In this scenario, falls are recognized as a relevant indicator of frailty and functional impairment, constituting one of the leading causes of morbidity, loss of independence, and risk of institutionalization [11].

Falls among older adults represent a major public health concern due to their multiple short- and long-term consequences, including fractures, balance disturbances, and injuries requiring prolonged recovery processes. Beyond their physical impact, falls also generate significant psychological effects, such as fear of falling, kinesiophobia, progressive social isolation, and increased functional dependence [12]. Collectively, these physical, psychological, and social consequences create a cycle of progressive decline that negatively affects the quality of life of older adults. In response to this scenario, governmental organizations have developed various preventive strategies aimed at maintaining functional capacity and reducing fall risk among older adults [13].

Among these interventions, multicomponent therapeutic exercise currently represents the primary preventive approach, integrating muscle strength training, balance, gait, flexibility, and functional tasks oriented toward activities of daily living. This type of program has demonstrated positive effects in reducing fall risk, improving physical performance, and preserving functional independence, positioning itself as the standard intervention across multiple community and clinical settings [1,11].

However, despite their demonstrated effectiveness, the implementation of traditional exercise programs presents relevant limitations in real-world care settings. Several studies have reported challenges related to sustained adherence over time, decreasing motivation in response to repetitive routines, limited immediate feedback on performance, and constraints in the program’s ability to dynamically adapt to individual changes in performance, preferences, and functional status among older adults [11]. These operational barriers may compromise therapeutic continuity and, consequently, the magnitude of the benefits achieved.

In this context, considering these limitations, virtual reality (VR) has emerged as a tool with the potential to complement and expand traditional therapeutic exercise strategies. Its incorporation into geriatric rehabilitation not only enables the reproduction of motor tasks oriented toward falls prevention, but also allows for the simultaneous integration of cognitive, sensory, and emotional demands within interactive and safe environments [14,15]. Unlike conventional programs, in which exercise progression and adaptation typically rely primarily on clinical judgment and periodic adjustments, VR enables continuous modulation of difficulty levels, immediate performance feedback, and real-time adjustment of task variables according to the user’s response.

The main contribution of VR lies in its capacity to provide personalized therapeutic experiences through the controlled manipulation of variables within the virtual environment, such as stimulus speed, motor complexity, attentional demands, required range of motion, and level of visual or auditory support. This level of personalization enables real-time adjustment of task intensity and complexity in response to user performance, fostering an optimal level of challenge that promotes neuroplasticity, motor learning, and active engagement in the rehabilitation process [16,17]. In contrast to traditional exercise programs, where individualization may be limited by group-based structures or constraints in human resources, VR allows for interventions that are simultaneously standardized and adaptive, maintaining protocol consistency without compromising clinical sensitivity.

Several studies have demonstrated its effectiveness in falls prevention and in improving functional performance, coordination, and movement confidence, thereby contributing to the physical and emotional well-being of older adults [15,18,19]. Furthermore, exergames provide a motivating, engaging, and adaptable environment tailored to individual capacities, promoting therapeutic adherence and sustained participation in exercise programs [14,19,20].

Although the literature supports the use of immersive virtual reality in older adults, the overall strength of the available evidence remains moderate to low. This is mainly due to methodological limitations, such as small sample sizes, heterogeneity in intervention protocols, and the lack of long-term follow-up, which highlights the need for further research to strengthen and consolidate these findings [21,22].

The present study focuses on determining the feasibility and implementation of a protocol using immersive virtual reality (exergames) for fall prevention in older adults at risk of frailty. This approach incorporates the development of standardized protocols while maintaining an individualized perspective that considers the specific needs and characteristics of each older adult. In this way, the interventions become consistent and reproducible without compromising clinical flexibility.

In addition, the protocol considers relevant constraints faced by health care teams, such as limited time and resources, by offering an alternative that complements traditional therapies and contributes to enhancing their effectiveness through an evidence-based intervention. In line with this rationale, the present work aims to implement an immersive virtual reality protocol focused on fall prevention, integrating new technologies into functional rehabilitation processes.

Accordingly, this protocol establishes the theoretical foundations, clinical criteria, technical procedures, and evaluation parameters required to standardize its application, ensuring both the safety of older adults and the therapeutic effectiveness of the intervention.

In line with the background described above, the present study proposes the following hypotheses:

Primary Hypothesis

**H1:**  *Older adults at risk of frailty who participate in the intervention will show a significant reduction in fall risk, as evidenced by an improvement in Timed Up and Go (TUG) test performance, compared with the control group.*

Secondary Hypotheses

**H2:**  *Older adults in the experimental group will exhibit a significant reduction in concern about falling, as measured by the Falls Efficacy Scale–International (FES-I), compared with the control group.*

**H3:**  *Older adults receiving the intervention will demonstrate a significant increase in balance-related confidence, as assessed by the Activities-specific Balance Confidence (ABC) Scale, compared with the control group.*

**H4:**  *The experimental group will show a significant improvement in physical performance, reflected by higher scores on the Short Physical Performance Battery (SPPB), including static balance, gait speed, and chair stand performance, compared with the control group.*

**H5:**  *Older adults in the experimental group will maintain handgrip strength, as assessed by handgrip dynamometry, compared with the control group, without evidence of significant post-intervention decline.*

## 2. Materials and Methods

### 2.1. Study Design

This study adopts a quantitative, applied approach with a quasi-experimental pre–post intervention design, aimed at evaluating the feasibility and preliminary effects of a virtual reality-based intervention (exergames) on functional and perceptual outcomes in older adults at risk of frailty [23].

The intervention will be implemented in a community-based setting within the primary health care (PHC) model, in accordance with the ethical and clinical safety guidelines established by the Chilean Ministry of Health (Decree with Force of Law No. 1, 2005).

### 2.2. Study Setting and Participants

Older adults enrolled in programs aimed at maintaining comprehensive health for older populations within the primary health care (PHC) system, particularly those affiliated with the More Self-Reliant Older Adults program (+AMA) in the city of San Pedro de la Paz, Biobío Region, Chile, will be invited to participate in the implementation of this protocol. The +AMA program is a nationwide intervention implemented by municipal health departments across Chile and is designed to promote self-reliance, self-care, and active ageing among adults aged 60 years and older [24]. The groups participating in this program comprise individuals with diverse functional levels and health conditions, including both autonomous older adults and those in a pre-frail condition or at risk of frailty, thereby allowing access to a heterogeneous and representative community-based population.

The selection of +AMA program groups and other older adult groups affiliated with PHC services is based on their operational feasibility for protocol implementation. These groups are characterized by stable organizational structures and regular participant attendance, which facilitates the delivery of the intervention. Access to participants will be managed through a collaboration agreement with the Municipal Health Department (Departamento de Salud Municipal, DAS) of San Pedro de la Paz. This agreement will define the mechanisms for coordination, dissemination, and execution of the protocol within the designated community settings, ensuring appropriate collaboration between health care teams and the implementation of the planned activities.

Allocation to experimental or control conditions will be conducted at the group level, based on pre-existing community groups. This approach aims to prevent contamination between participants, preserve the natural group structure of PHC workshops, and enhance ecological validity. Given the community-based nature of the intervention, individual randomization was not feasible. Baseline comparability between groups will be examined prior to outcome analyses.

### 2.3. Sample Size

**Figure 1:**
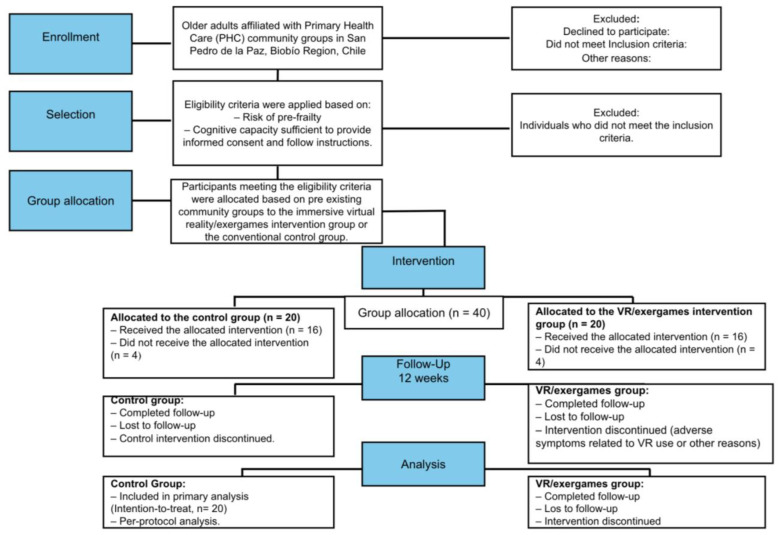
Adapted CONSORT flow diagram of the intervention under the immersive virtual reality protocol.

The sample size (*n* = 40) was calculated a priori for a mixed repeated-measures design with two groups (control vs. experimental) and two assessment time points (pre- and post-intervention). The calculation assumed a significance level of α = 0.05, a statistical power (1 − β) of 0.80, and an expected small-to-moderate effect size (f = 0.25), with an assumed within-participant correlation of 0.5 and a nonsphericity correction of ε = 1.0. Under these assumptions, a minimum of 34 participants was required, evenly distributed as 17 individuals per group. Considering the potential for participant attrition and to ensure an adequate sample for pre- and post-intervention analyses, an attrition rate of approximately 15–20% was anticipated. Therefore, a total of 40 older adults will be recruited, with 20 participants allocated to the experimental group and 20 to the control group. The flow of participants through each stage of the study will be presented using a adapted CONSORT flow diagram adapted for quasi-experimental studies (Figure 1).

### 2.4. Participant Recruitment

Participant recruitment will be conducted across the different groups affiliated with primary health care (PHC) services in the municipality of San Pedro de la Paz, Biobío Region, Chile.

The recruitment process will be carried out by trained research support staff. Initial contact will be made by telephone with the representatives of the participating groups, followed by the coordination of in-person visits to the locations where group activities take place. During these visits, older adults will be invited to participate in the study and will be provided with clear and comprehensible information regarding the study objectives, the general characteristics of the virtual reality (VR) intervention, its duration, and the associated assessment procedures. Emphasis will be placed on the voluntary nature of participation and on the participants’ right to withdraw from the study at any time without consequences.

Older adults who express interest in participating will be assessed to verify compliance with the established eligibility criteria. Final inclusion in the study will occur only after the informed consent form has been signed, ensuring adherence to the ethical principles of autonomy, confidentiality, and safety for older participants.

To safeguard confidentiality and anonymity, participant identification data will be coded using alphanumeric identifiers. These codes will also be used to differentiate between pre- and post-intervention measurements.

### 2.5. Inclusion and Exclusion Criteria—Selection Criteria

The inclusion and exclusion criteria for older adults participating in this study are detailed in Table 1.

### 2.6. Ethical Considerations

#### 2.6.1. Informed Consent and Ethical Principles

Final inclusion in the study will occur only after participants have signed the informed consent form, which will ensure adherence to the ethical principles of autonomy, confidentiality, and safety of older adults. The informed consent document will clearly describe the study objectives, intervention and assessment procedures, potential risks associated with the use of immersive virtual reality (IVR), the voluntary nature of participation, and the right to withdraw at any time without consequences for their usual care.

The protocol was reviewed and approved by the Scientific Ethics Committee of Universidad de Las Américas (Chile) under approval code CEC_PI_2025013. The study will be conducted in accordance with the principles of the Declaration of Helsinki and current national regulations.

#### 2.6.2. Confidentiality and Data Protection

All information collected during the study will be safeguarded under the responsibility of the principal investigator. To ensure the confidentiality and anonymity of participants, identifying data will be coded using an alphanumeric identification system. This coding procedure will allow differentiation between pre- and post-intervention measurements without compromising participants’ identities.

Physical documentation will be stored in a secure, locked location, and digital data will be maintained on password-protected devices and encrypted storage systems. All study-related documents will be retained for a period of five years following study completion, after which they will be securely destroyed in accordance with current regulations.

#### 2.6.3. Safety During the Intervention

The implementation of the immersive virtual reality (IVR) intervention will be conducted under strict clinical safety measures aimed at safeguarding the physical integrity and well-being of participants. Prior to the formal initiation of the program, a familiarization session will be conducted to provide standardized instructions regarding device use, characteristics of the virtual environment, and potential symptoms associated with the immersive experience, such as dizziness, nausea, or fatigue.

During each session, the professional team will ensure continuous supervision of participant performance and tolerance, monitoring for signs of postural instability, physical discomfort, or disorientation. In the event of any adverse event, the fall and emergency response protocol will be immediately activated, establishing clear procedures for first aid, notification, and referral to emergency services when required.

Additionally, symptoms associated with virtual reality use (cybersickness) will be systematically assessed using validated instruments, allowing for the documentation of their frequency, intensity, and relationship to the intervention. This structured monitoring strategy contributes to ensuring participant safety and to rigorously documenting adverse events as part of the study’s feasibility indicators.

#### 2.6.4. Digital Divide and Equity in Participation

To minimize potential bias associated with the digital divide, the program incorporates a structured technological “familiarization” phase prior to the formal initiation of the intervention. This stage includes progressive, supervised, and guided exposure to the virtual reality device, encompassing basic headset training, comprehension of task instructions, and sensorimotor adaptation to the immersive environment. The implementation of this foundational phase aims to standardize baseline participation conditions, reducing differences derived from prior levels of digital literacy.

This strategy contributes to decreasing variability attributable to uncontrolled technological factors, thereby strengthening internal validity. Additionally, it helps mitigate potential adverse effects, such as anxiety or insecurity related to technology use, which may interfere with performance during the initial sessions. Prior experience with digital devices is explicitly not considered an exclusion criterion, ensuring inclusive access and alignment with health equity principles within primary health care settings.

### 2.7. Intervention Procedures

Virtual reality (VR) is an interactive technology that enables users to experience a sense of immersion within digitally generated three-dimensional environments through devices integrating head-mounted displays (HMDs), motion sensors, and audiovisual feedback systems [15]. Its primary purpose is to simulate controlled scenarios in which objects and environmental variables can be manipulated, allowing spatial, temporal, and sensorimotor interactions that elicit specific motor and cognitive responses.

Among its modalities, immersive virtual reality (IVR) provides a first-person experience through head-mounted displays equipped with independent screens for each eye, head tracking systems, and handheld controllers that enable direct interaction with virtual objects. This configuration promotes a high sense of presence, defined as the subjective perception of physically being within the digital environment [14].

Mixed virtual reality integrates elements of virtual reality and augmented reality, allowing the superimposition of interactive virtual stimuli onto the real physical environment through camera-based systems and spatial sensors [15]. This modality enables hybrid interaction while maintaining real-world spatial references, which is particularly relevant in interventions targeting balance and functional mobility [25,26].

Scientific evidence indicates that balance exercises delivered through virtual environments generate beneficial effects in older adults. These effects are attributed to the simultaneous integration of motor, sensory, and cognitive demands, which stimulate motor learning processes and postural adaptation [27]. Virtual reality (VR)-based active video games (exergames) introduce an additional component of progressive challenge and real-time feedback. From a physical perspective, they require activation of large muscle groups through functional reaching, weight shifting, and controlled displacement. At the cognitive level, they involve attentional processing, stimulus discrimination, and decision-making. Immediate feedback allows real-time adjustment of motor execution, facilitating the correction of postural patterns and the optimization of dynamic balance [28,29]. At the emotional level, progressive exposure to balance tasks within a controlled environment has been associated with a reduction in fear of falling and an increase in movement confidence. These effects may be explained by improvements in perceived self-efficacy and the safe repetition of functional tasks [30].

Additionally, the implementation of VR in primary health care (PHC) settings may offer operational advantages, as it enables the delivery of structured therapeutic programs within limited physical spaces, thereby optimizing material and human resources [29]. However, there is currently limited standardization of specific protocols for the application of virtual reality in older adults within health care contexts, which justifies the development of the present procedure.

#### 2.7.1. Technical Specifications of the Intervention Platform

**Figure 2:**
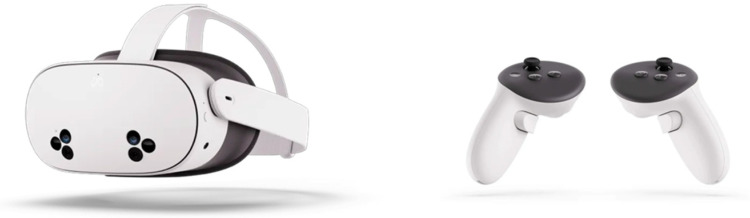
Oculus Meta Quest 3s headset and controllers.

The implementation of this protocol will be based on the use of immersive and mixed virtual reality technologies through the Oculus Meta Quest 3S device (Meta Platforms, Inc., Menlo Park, CA, USA). This device allows users to engage in both immersive virtual reality and mixed reality experiences using a head-mounted display (HMD) with a resolution of 1832 × 1920 pixels per eye, a refresh rate of 120 Hz, and a field of view of 96° horizontal and 90° vertical, with an integrated stereo sound system [31]. The Meta Quest 3s includes two Touch controllers (Figure 2); however, it also incorporates hand tracking and head tracking systems, enabling natural interaction with the virtual environment without the permanent need for physical controllers. In terms of ergonomics, the device weighs approximately 512 g and features an adjustable head strap for cranial stability, as well as compatibility with prescription lenses, characteristics that enhance comfort and adaptability during prolonged sessions.

Additionally, the Meta Horizon mobile application includes a screen mirroring function, allowing real-time projection of the visual experience generated by the virtual reality headset. This feature facilitates participant performance monitoring by the professional team, enabling early detection of difficulties, immediate feedback, and the promotion of group engagement within the intervention process.

The selection of this device is based on its capacity to generate controlled environments through immersive and mixed reality experiences, enabling synchronous interaction between the older adult, the digital environment, and the physical space. Compared with earlier-generation devices, such as the Oculus Meta Quest 2, recent models offer improvements in latency, refresh rate, and spatial tracking, which may reduce the incidence of symptoms such as nausea, dizziness, or disorientation (cybersickness) and optimize intervention feasibility.

#### 2.7.2. Pre-Intervention Assessment (Phase 1)

As part of the program implementation, a pre-intervention assessment will be conducted, focusing on the evaluation of multiple functional and perceptual components. Vestibular-related symptoms will be assessed using the Dizziness Handicap Inventory (DHI). Physical performance will be evaluated through handgrip dynamometry, while functional mobility will be assessed using the Timed Up and Go (TUG) test and the Short Physical Performance Battery (SPPB). Functional status will be measured using the Barthel Index and the Instrumental Activities of Daily Living scale by Hernández and Neumann. The psychological dimension associated with falls will be evaluated using the Falls Efficacy Scale–International (FES-I) and the Activities-specific Balance Confidence (ABC) Scale.

A maximum period of two weeks will be established for the completion of all baseline assessments. Following this period, minimum eligibility criteria for participation in the intervention will be confirmed prior to the initiation of the immersive virtual reality-based intervention.

Regardless of group allocation (control or experimental), each intervention session will follow the structured schedule described in Table 2, with a total duration of 60 min (including preparation, warm-up and cool-down phases, exercises, and rest periods). According to the Manual for Fall Prevention in Older Adults [32], sessions should focus on exercises targeting muscle strengthening, static and dynamic balance, weight-shifting activities, stretching exercises, functional reaching tasks, strength training, and postural control responses, including righting, support, and balance reactions. In addition, the intervention will be adapted according to participants’ interests, preferences, difficulty level, and individual functional profiles.

During the third week of the pre-intervention phase, an introductory session on virtual reality will be conducted, aimed at the progressive familiarization of participants with the technology. During this period, supervised exploratory sessions will be carried out, allowing older adults to become acquainted with the devices, experience their basic functioning, and clarify any questions. In addition, psychoeducational information will be provided regarding the benefits of virtual reality in rehabilitation processes and functional maintenance, with the aim of promoting adherence and reducing potential concerns associated with technology use among this age group.

**Figure 3:**
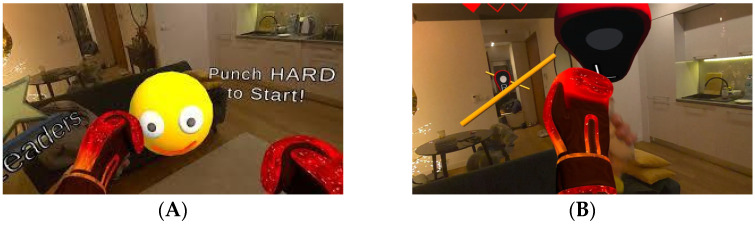
Views of the Boxing Game mode in Oculus Meta Quest 3s. (**A**) First-person view during gameplay in mixed reality in a static position. (**B**) First-person view during gameplay in mixed reality in dynamic movements.

In the virtual reality workshops, the application Boxing Game, available on the Oculus platform, will be implemented. This tool incorporates mini games in a mixed virtual reality format that require participants to intercept virtual objects either from a static position or through controlled movements (Figure 3). The selection of this application was based on a systematic analysis of various applications and exergames, considering criteria such as accessibility, clarity of instructions, low initial complexity, and the ability to adjust variables such as stimulus speed and frequency. These characteristics allow the activity to be adapted to the functional level of older adults and to support therapeutic progression. During this session, designated as Session 0, monitoring adverse symptomatology associated with the use of virtual reality is essential to ensure continuity within the program.

#### 2.7.3. Monitoring of Adverse Events

To ensure the relevance, safety, and acceptability of the virtual reality (VR) program and the targeted interventions among older adults, the following technical and user-centered criteria are established:

1. Environmental Preparation:

An appropriate physical space is essential to promote concentration and minimize risks. In this regard, it is necessary to: Ensure an obstacle-free environment that does not compromise physical safety during the experience. Guarantee adequate lighting, comfortable temperature, and proper ventilation to facilitate participation of older adults, and minimize environmental noise to enhance immersion during the session.

2. Device Placement and Adjustment:

The responsible professional must: Demonstrate how to wear the headset and use the controllers. Adjust the device individually to ensure comfort, stability, and proper visualization of the virtual environment. Verify that the user maintains a safe posture throughout the experience. For participants who wear prescription glasses, an appropriate spacer accessory will be incorporated to extend the headset.

3. User Preparation:

Before initiating the session, participants must be provided with clear and comprehensible information, delivered in verbal, visual, and auditory formats. Specifically, the following is recommended: Participants will undergo monitoring of physiological parameters, particularly blood pressure, at the beginning and at the end of the session. Additionally, the functioning of the virtual reality (VR) device and the characteristics of the experience must be explained precisely. Participants should be informed about possible sensations that may be experienced (e.g., immersion, mild dizziness, or initial disorientation). Any questions should be addressed and concerns managed throughout the virtual experience.

4. Monitoring During the Session:

Continuous supervision is essential to prevent adverse events. Accordingly, it is necessary to: Continuously observe the user’s physical and emotional responses. Identify signs of discomfort, anxiety, fatigue, or distress (e.g., nausea or postural instability) associated with cybersickness. Immediately interrupt the session if the participant requests it or if any adverse reaction is detected.

5. Post-Session Evaluation:

At the conclusion of the intervention session, a comprehensive evaluation should be conducted, including the collection of the user’s subjective perception of the experience. Preliminary assessment of the impact of VR on variables related to fall prevention (e.g., balance, confidence in gait, or reaction time). Administration of the Simulator Sickness Questionnaire (SSQ) and systematic documentation of clinical observations by the multidisciplinary professional team.

6. Action Protocol for Falls and Emergencies

This protocol establishes the procedures designed to ensure a timely, effective, and efficient response to adverse events occurring during the implementation of project activities. Its primary purpose is to safeguard the physical integrity and well-being of participants by ensuring high-quality care through the appropriate use of available human and material resources.

The adverse and/or sentinel events considered within this protocol include, but are not limited to, falls, loss of consciousness, wounds, traumatic injuries, and any other situation that may compromise the participant’s physical stability.

The professional team will be responsible for activating and implementing the action protocol in the event of a critical or adverse incident, as well as for carrying out the corresponding notification in accordance with institutional guidelines.

In the event of an incident, the following procedures must be followed:

(a) A member of the multidisciplinary healthcare team shall provide first aid to the affected participant, assessing their general condition and ensuring basic stabilization measures.
(b) Simultaneously, another team member shall contact the appropriate emergency service, clearly and accurately reporting the nature of the incident, the participant’s condition, and the exact location.
(c) The professional in charge shall communicate with the participant’s registered emergency contact, informing them of the incident, the actions taken, and any potential referral to a healthcare facility.
(d) Subsequently, the incident must be formally reported to the project coordination team, with written documentation completed in accordance with established administrative procedures.

These criteria are aligned with the protocols used by healthcare services, specifically within Family Health Centers (CESFAM), primary care facilities in which older adults actively participate as a priority user population.

#### 2.7.4. Virtual Reality-Based Falls Prevention Workshop (Intervention) (Phase 2)

##### VR-Based Program Implementation Sessions

The intervention sessions corresponding to the Virtual Reality Program for Older Adults in Primary Health Care (PHC), aimed at falls prevention, will be implemented in accordance with the guidelines established in the Information Guide for Therapeutic Interventions with Immersive Virtual Reality (INVIRTUE) [33]. The clinical objective of the program is to reduce fall risk and maintain functional capacity within the framework of strategies for preventing functional decline in older adults. The intervention will have a total duration of twelve weeks, with a frequency of two sessions per week and an approximate duration of 60 min per session. The process will be supervised by an interdisciplinary team composed of occupational therapists and speech-language pathologists, who will provide continuous clinical monitoring, personalized feedback, and timely management of any adverse symptoms associated with the use of immersive virtual reality (iVR), such as dizziness, nausea, or disorientation.

Prior to the initiation of the program, an introductory session will be conducted to familiarize participants with the technology. During this session, explanatory audiovisual content will be presented, information regarding the therapeutic objectives will be provided, and a practical demonstration of device use will be performed. Participants’ questions will be addressed, and potential concerns related to the immersive experience will be discussed.

For the deployment of the intervention, appropriate preparation of the physical environment will be essential, ensuring a spacious, safe area free of obstacles. During each session, the instructor will activate the device and establish the necessary connection to project the experience onto an external screen, allowing the remaining group members to observe the activity and participate interactively through verbal feedback.

Subsequently, each participant will directly engage with the immersive virtual reality environments under constant supervision by the professional team, ensuring safety conditions, therapeutic support, and systematic monitoring of intervention tolerance.

#### 2.7.5. VR-Based Program Sessions

Following successful completion of this familiarization phase, the 12-week intervention program will be implemented using the KINESIX XR^®^ will constitute the core therapeutic component of the 12-week intervention phase, available in the official Meta Store, will be used. This application was developed by KINESIX, a neurotechnology company based in Montreal, Canada, specializing in the development of immersive digital therapeutics. Its programs are supported by clinical evidence and designed by therapists specialized in neurorehabilitation [34]. This constitutes the structured therapeutic platform for progressive motor and functional training throughout the intervention period.

Based on an analysis of the exercises available on the platform, the “360° Gym” module will be initially selected. This module focuses on the execution of functional movements, including upper-limb mobilization in multiple directions, lower-limb movements, trunk displacement and control, and weight-transfer exercises. This selection aims to promote global motor patterns and improve coordination and postural control in participants.

**Figure 4:**
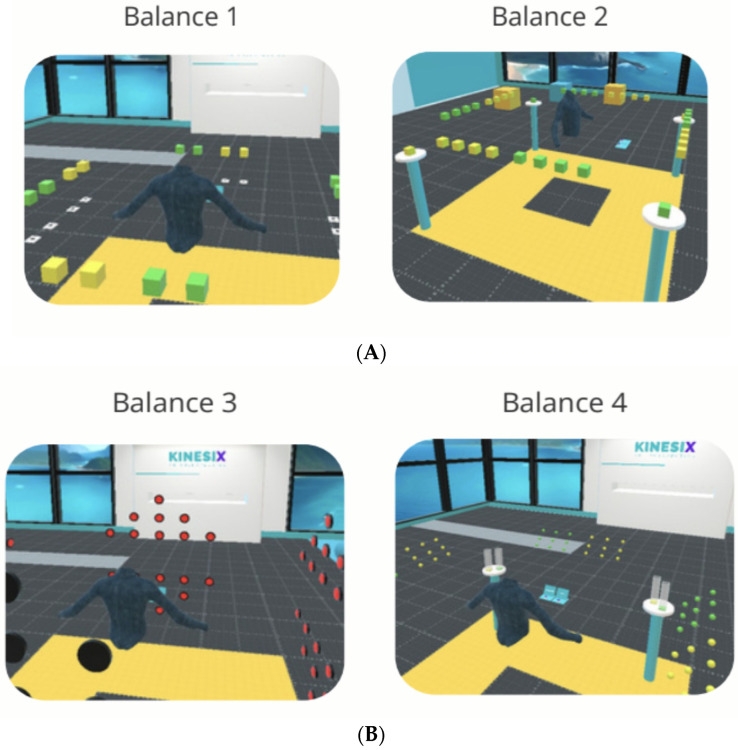
Views of “Gym 360” exercises of Balance. (**A**) Point of view professional exercises of balance 1 and 2. (**B**) Point of view professional exercises of balance 3 and 4.

In the virtual context of Balance Exercise 1, participants are required to reach for specific stimuli represented by green and yellow blocks. These blocks must be grasped and placed into previously assigned shadow targets corresponding to the same color. Through this activity, functional reaching, weight shifting, laterality, midline crossing, and balance reactions are trained, thereby promoting postural control and motor integration (Figure 4). Subsequently, in Balance Exercise 2, participants must move within the virtual environment to reach the green and yellow blocks and place them on designated tables according to the corresponding color. This second activity incorporates greater demands on dynamic balance, locomotion, and spatial coordination, progressively increasing motor complexity compared to the previous exercise (Figure 4).

In Balance Exercise 3, participants will receive visual stimuli, specifically lights, which they must deactivate by following a previously established pattern. This activity will prioritize lateral and anteroposterior weight shifts, controlled weight unloading, and functional reaching tasks, with the aim of promoting dynamic postural adjustments and standing balance control (Figure 4). In Balance Exercise 4, a cognitive component will be incorporated alongside movement. Each participant will be required to reach for colored balls and place them into a container that follows a specific pattern assigned to a given color (green or yellow). This task integrates functional mobility, hand–eye coordination, and cognitive processing related to color discrimination and associative matching (Figure 4).

**Figure 5:**
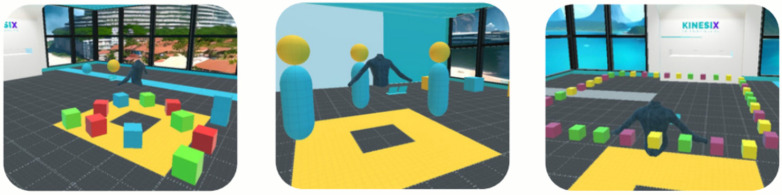
Views of exercises on Activities of Daily Living (ADL) on KINESIX XR. Point of view professional on vestibular exercises.

In addition to balance and strengthening components, the intervention program will incorporate specific exercises targeting vestibular system training, including visual tracking activities, head rotations, directional changes during ambulation, body turns, and both static and dynamic balance tasks (Figure 5). These activities are designed to progressively stimulate sensory integration mechanisms and postural control processes, with the aim of optimizing stability and reducing fall risk among older adults within an immersive virtual reality environment.

**Figure 6:**
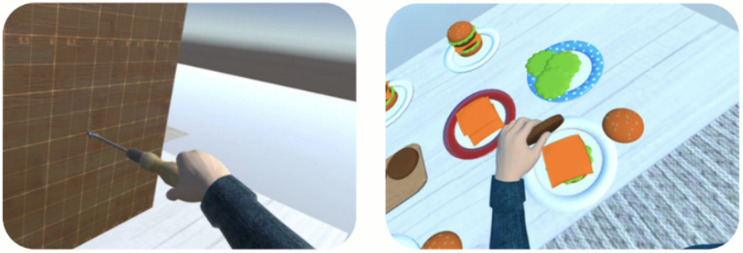
Views of exercises on Activities of Daily Living on KINESIX XR. First-person view exercises of AVD.

Finally, all sessions will include closing exercises focused on activities of daily living (ADLs). The purpose of these activities is to facilitate the transfer of the skills trained to real-world contexts, enabling older adults to integrate and apply the competencies acquired in virtual environments to everyday situations (Figure 6).

Each of these activities will allow real-time adjustment of the number and speed of stimuli, as well as execution time, according to the individual characteristics of the participants, under the supervision of the professional team. The incorporation of these customizable features within digital applications enhances adherence to the intervention, as it enables task personalization based on goals centered on each individual’s needs and specific functional profile. Additionally, performance monitoring will be conducted in real time through tracking and feedback mechanisms integrated into the KINESIX XR^®^ system.

In accordance with the previously developed theoretical and empirical foundations, the virtual reality sessions designed for the fall prevention program in older adults would be structured according to the criteria presented in Table 2.

Following the immersive virtual reality intervention sessions, each participant will be assessed for adverse symptoms related to technology use (cybersickness), to ensure user safety, provide feedback to the research team, and prevent potential situations that could compromise participant well-being [35].

#### 2.7.6. Conventional Fall Prevention Workshop (Intervention) (Phase 2)

The conventional-format intervention sessions will receive instructions from a professional team composed of an occupational therapist and a speech-language therapists, who will guide participants through exercise workshops focused on muscle strengthening, static and dynamic balance training, weight-shifting activities, stretching exercises, functional reaching tasks, strength training, and postural control responses, including righting, support, and balance reactions, using a traditional, non-technology-based approach.

**Figure 7:**
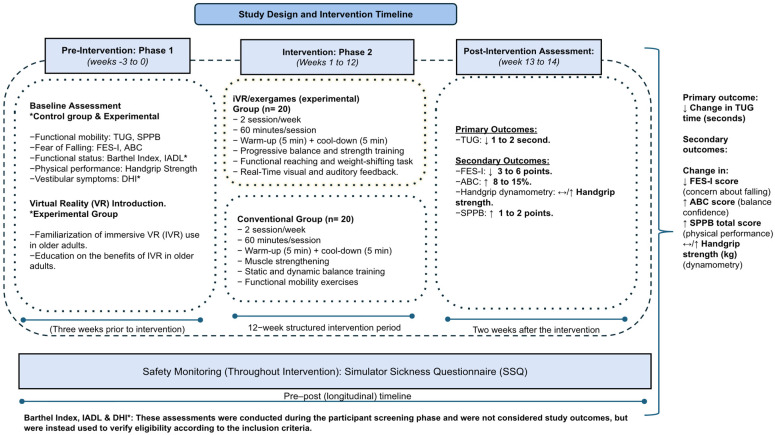
A quasi-experimental Virtual Reality-Based Program for Fall Risk Reduction in Older Adults in Primary Health Care.

These workshops will follow the same distribution schedule as the experimental intervention, consisting of two sessions per week, each lasting 60 min. Sessions will be structured into a warm-up phase, a main exercise phase, and a cool-down phase, with scheduled rest periods distributed between exercises; the only difference will be the modality of the exercise program. The process for both the experimental and conventional interventions is illustrated in Figure 7.

### 2.8. Statistical Analysis

Data analysis will be performed using IBM SPSS Statistics software, version 30.0. Prior to analysis, data cleaning and coding procedures will be conducted to verify the absence of errors, outliers, or missing values. All statistical analyses will be carried out using a significance level of *p* < 0.05 and a 95% confidence interval.

At an initial stage, a descriptive and exploratory analysis will be conducted to characterize the sample and examine the distribution of the study variables. Sociodemographic variables (including age, sex, educational level, and comorbidities) will be summarized using absolute and relative frequencies for categorical variables, and measures of central tendency and dispersion (mean, median, standard deviation, and interquartile range) for continuous variables. Normality will be assessed using the Shapiro–Wilk test. Based on distributional assumptions, between-group comparisons will be performed using the independent-samples *t*-test for normally distributed continuous variables, the Mann–Whitney U test for ordinal variables or continuous variables with non-normal distributions, and the chi-square test for nominal variables.

To evaluate the effects of the virtual reality intervention, a two-way repeated-measures ANOVA will be conducted to test group-by-time effects (group × time). The primary analysis will follow the intention-to-treat (ITT) principle. A minimum of 20 imputed datasets will be generated, and this number may be increased depending on the proportion of incomplete data. Effect sizes will be estimated using partial eta-squared (ηp^2^) and interpreted as small (0.01), medium (0.06), or large (0.14) [36].

To control for inflation of type I error across secondary endpoints, *p* values for the prespecified secondary outcome analyses will be adjusted using the Holm–Bonferroni sequential procedure. This method was selected because it appropriately controls the family-wise error rate in the presence of multiple comparisons while being less conservative than the traditional Bonferroni correction. The correction will be applied to the secondary outcomes FES-I, ABC, SPPB, and handgrip strength. For the primary outcome, unadjusted *p* values will be reported. For the secondary outcomes, both unadjusted and Holm-adjusted *p* values will be presented, together with effect sizes and 95% confidence intervals [37].

To control for inflation of type I error across the secondary outcomes, the Holm–Bonferroni sequential correction procedure will be applied to FES-I, ABC, SPPB, and handgrip strength [38,39]. This method was selected because it appropriately controls the family-wise error rate in the presence of multiple comparisons while being less conservative than the traditional Bonferroni correction [38,39]. For the primary outcome, unadjusted *p* values will be reported, whereas for the secondary outcomes both unadjusted and adjusted *p* values will be presented. Regarding statistical power, because the sample size was determined primarily based on the primary outcome, the secondary outcomes will be considered supportive and exploratory. With a statistical power of 80%, the study is expected to be more sensitive to large effects, whereas small effects in the secondary outcomes may not be detectable. Therefore, these findings will be interpreted with caution.

### 2.9. Complementary Analyses

To assess the feasibility of implementing the protocol within the primary health care setting, an analysis of feasibility indicators will be conducted using descriptive statistics and graphical representations:

#### 2.9.1. Recruitment Rate

The recruitment rate will be calculated as the percentage of individuals who agree to participate in the study relative to the total number of individuals invited. This indicator will allow the feasibility of the recruitment process and the effectiveness of the invitation strategies to be assessed. The target sample size (*n* = 40) was determined a priori for a mixed repeated-measures design with two groups (control vs. experimental).

#### 2.9.2. Retention Rate

The retention rate is defined as the percentage of participants who complete all planned sessions and assessments relative to the total number of individuals who initiate the study. This indicator will be used to evaluate participant retention and continuity throughout the protocol. Based on the planned sample size, a minimum retention rate of 80% is expected for both the experimental and control groups (*n* = 32).

#### 2.9.3. Reported Adverse Events

Following each immersive virtual reality session, participants will be assessed for the presence of signs and symptoms associated with technology use (cybersickness), such as dizziness, nausea, headache, disorientation, or general discomfort. The systematic recording of these events will ensure participant safety, provide feedback to the research team, and help prevent situations that could compromise participant well-being. All adverse events will be classified according to type, severity, and their relationship to the intervention, and will be documented in a dedicated registry for subsequent analysis.

#### 2.9.4. Intervention Adherence

Intervention adherence is defined as the percentage of intervention sessions effectively completed by each participant relative to the total number of sessions planned in the protocol. This indicator will be used to assess the level of compliance and active participation of participants in the intervention. Based on the study design, a minimum adherence rate of 80% is expected in both the experimental and control groups (*n* = 32).

## 3. Expected Results

Pre- and post-intervention assessments will be conducted across functional, physical, and emotional domains. These assessments will be administered by trained health care professionals who will not be involved in the delivery of the interventions, thereby ensuring the independence of the evaluation process. In both the experimental and control groups, a system of rotating evaluators will be implemented to enhance objectivity and minimize potential assessment bias.

In addition, an assessment blinding process will be implemented. Scoring batches will be randomized, and standardized training and inter-rater reliability protocols will be established. Evaluators will work with alphanumeric identifiers (IDs) and a coded temporal marker, so that pre- and post-intervention conditions will not be identifiable during the data analysis process.

The primary outcome of this study is a reduction in fall risk among older adults at risk of frailty, as measured by the Timed Up and Go (TUG) test. This change is expected to be more pronounced in the experimental group compared with the control group.

As secondary outcomes, the experimental group is expected to demonstrate a significant reduction in concern about falling, as measured by the Falls Efficacy Scale–International (FES-I), and an increase in balance-related confidence, assessed using the Activities-specific Balance Confidence (ABC) Scale. Improvements in physical performance are also anticipated, including static balance, gait speed, and lower-limb strength, as evaluated through the components of the Short Physical Performance Battery (SPPB), including the chair stand test. In addition, maintenance of handgrip strength, assessed through handgrip dynamometry, is expected. Taken together, these outcomes are anticipated to be more pronounced in the experimental group, reflecting the potential effects of the proposed intervention.

### 3.1. Primary Outcomes

*Fall Risk:*

Fall risk will be operationalized using the Timed Up and Go (TUG) test, a performance-based measure of functional mobility and dynamic balance that records the time (s) required to stand up from a chair, walk 3 m, turn, return, and sit down again [40]. The TUG has been widely validated across different countries and is routinely used to estimate fall risk in community-dwelling older adults.

For the assessment, participants will be seated with their back supported against the backrest and both feet placer firmly on the floor. On the command “go”, they will stand up without using their arms for support, walk at their usual and safe pace to a cone positioned 3 m away, tur demonstration will be provided prior to testing to ensure proper understanding of the procedure [41].

For clinical interpretation, after the 12-week intervention, a TUG time >13.5 s will be considered indicative of increased fall risk in community-dwelling older adults. In addition, an individual pre–post improvement of ≥2.3 s will be considered to exceed measurement error (MDC90), supporting interpretation of a “true” change. Evidence also supports an association between longer TUG times and falls in older adults, although cut-off values and predictive performance may vary across settings and populations [42].

### 3.2. Secondary Outcomes

#### Concern About Falling

*Falls Efficacy Scale–International (FES-I):*

The Falls Efficacy Scale–International (FES-I) was developed to provide a comprehensive measure of concern about falling, incorporating both physical and social factors, while ensuring suitability for cross-cultural applications. Available evidence strongly supports its use in older adult populations [43]. The FES-I consists of 16 items in which participants rate their level of concern about the possibility of falling in different situations, using a scale ranging from 1 (not at all concerned) to 4 (very concerned). A total score greater than 23 is commonly considered indicative of elevated or clinically relevant concern regarding fall risk [44].

In the present study, is expected on experimental group to show a 3-to-6-point reduction in total FES-I scores, with a lower proportion of participants scoring > 23, reflecting a small-to-moderate effect size (d ≈ 0.40–0.60). This improvement would indicate enhanced perceived self-efficacy and reduced activity restriction due to fear of falling [44].

*Activities-specific Balance Confidence (ABC) Scale:*

The ABC questionnaire consists of 16 items and may be self-administered or applied by a trained evaluator. It is a widely used subjective measure designed to assess balance confidence and the degree of fear of falling experienced by an individual when performing various daily activities, such as moving around the home, ascending or descending stairs, getting into or out of a car, or bending down to pick up objects from the floor, among others [45]. Participants rate their confidence in performing each activity without losing balance or becoming unsteady, generating a total score expressed as percentage, with higher values indicating greater balance confidence.

In this study, changes in total ABC score will be analyzed to evaluate the effect of the intervention on balance-related confidence. A value ≥ 67% has been associated with lower fall risk in community-dwelling older adults and will be considered clinically relevant threshold. Improvements in total score, as well as an increase in the proportion of participants reaching value ≥ 67%, will be interpreted as reflecting enhanced perceived safety and functional confidence during activities of daily living. A small-to-moderate effect size (d ≈ 0.40–0.70) is anticipated,

*Short Physical Performance Battery (SPPB):*

The Short Physical Performance Battery (SPPB) includes three sequential performance-based tests administered over approximately 6–12 min: static balance (feet together, semi-tandem, and tandem), gait speed over 2 or 4 m, and the five-repetition chair stand tests [46].

The experimental group is expected to achieve a clinically meaningful increase of ≥1 point in the total SPPB score, with a small-to-moderate effect size (d ≈ 0.40–0.70), reflecting improved lower-extremity function and overall functional stability.

*Handgrip Dynamometry:*

Muscle strength can be assessed using various procedures; however, handgrip strength measurement has become one of the most used tools in epidemiological studies due to its ease of administration, high reliability, and low cost. These characteristics make it a relevant health biomarker, with wide applicability in both clinical settings and population-based research [47]. In Chile, a study conducted by Lera et al. evaluated 5255 adults aged 60 years and older, reporting muscle weakness cut-off points of ≤27 kg for men and ≤15 kg for women [48].

In the present study, handgrip strength will be measured at baseline and post-intervention to examine potential changes associated with the immersive virtual reality program. Stability or attenuation of decline in handgrip strength in the experimental group, compared with the control group, will be interpreted as reflecting preservation of overall muscular function within the context of fall risk prevention.

## Figures and Tables

**Table 1:** Inclusion and exclusion criteria.

Inclusion Criteria	Exclusion Criteria
○ Age ≥ 60 years.	○ Progressive neurological diseases or major neurocognitive disorders.
○ Functional status classified according to EFAM and the Barthel Index: autonomous older adults without risk or with mild risk of dependency.	○ Uncompensated sensory impairments.
○ Mild frailty according to Fried’s criteria or low handgrip strength (men < 27 kg; women < 16 kg).	○ Recent history of severe fractures or traumatic brain injury (TBI).
○ Timed Up and Go (TUG) test between 10 and 20 s. ○ Falls Efficacy Scale–International (FES-I) score between 20 and 40 points.	○ IMC ≥ 35.
○ Activities-specific Balance Confidence (ABC) Scale score ≥ 67%. ○ Dizziness Handicap Inventory (DHI) score ≥ 30 points. ○ Older adults who have not received interventions aimed at fall prevention or cognitive function stimulation for at least six weeks prior to study enrollment.	○ Decompensated heart disease or uncontrolled cardiac arrhythmias.
○ Provision of written informed consent.	
○ Adequate tolerance to visual and auditory stimuli.	

**Table 2:** This is a table of Virtual Reality-Based Program for Fall Risk Reduction in Older Adults in Primary Health Care.

Activities/Exercises	Time	Description
Equipment set-up and safety check	−Pre-start to +3 min from session onset	Brief instructions, device adjustment, verification of safe area, and confirmation of participant readiness.
Warm up	5 min.	Use of exercises based on the Fall Prevention Manual for Older Adults and the Wheel within the VIVIFRAIL Program (Categories B, C1, C2, and D)
Balance 1	5 min.	Balance exercises; trunk control exercises, Weight shifting and functional reaching.
Balance 2	8 min.	Balance exercises, trunk control exercises, dynamic balance, locomotion, spatial coordination.
REST/transition	3 min.	Rest.
Balance 3	8 min.	Balance exercises, trunk control exercises, dynamic balance, locomotion, spatial coordination.
Balance 4	5 min.	Balance exercises, trunk control exercises, dynamic balance, locomotion, spatial coordination. Functional mobility.
REST/transition	3 min.	Rest.
Vestibular	8 min.	Vestibular exercises, depending on the needs of the participating older adults.
ADL	5 min.	Daily life-based exercises.
COOL-DOWN	5 min.	Exercises according to the Fall Prevention Manual for Older Adults and VIVIFRAIL program, with emphasis on breathing technique, flexibility, and prevention of cybersickness symptoms.
“Total active intervention time: 60 min including preparation and rest periods.”		

VIVIFRAIL: Multicomponent Physical Exercise Program; Category B: Moderate functional limitation; Category C1: Frail; Category C2: Pre-Frail; D: Autonomous; ADL: Activities of Daily Living.

## Data Availability

Data will be available upon reasonable request from the corresponding author.

## Abbreviations

The following abbreviations are used in this manuscript:

RV	Virtual Reality
APS	Primary Health Care
+AMA	More Independent Seniors Program
EFAM	Functional Assessment of Older Adults
DHI	Dizziness Handicap Inventory
TUG	Timed Up and Go
SPPB	Short Physical Performance Battery
FES-I	Falls Efficacy Scale-International
ABC	The Activities-specific Balance Confidence Scale
SSQ	Simulator Sickness Questionnaire
INVIRTUE	Information Guide for Therapeutic Interventions with Immersive Virtual Reality
VIVIFRAIL	Multicomponent Physical Exercise Program
